# Natural Language Processing for Improved Characterization of COVID-19 Symptoms: Observational Study of 350,000 Patients in a Large Integrated Health Care System

**DOI:** 10.2196/41529

**Published:** 2022-12-30

**Authors:** Deborah E Malden, Sara Y Tartof, Bradley K Ackerson, Vennis Hong, Jacek Skarbinski, Vincent Yau, Lei Qian, Heidi Fischer, Sally F Shaw, Susan Caparosa, Fagen Xie

**Affiliations:** 1 Epidemic Intelligence Service Centers for Disease Control and Prevention Atlanta, GA United States; 2 Department of Research & Evaluation Kaiser Permanente Southern California Pasadena, CA United States; 3 Kaiser Permanente Bernard J. Tyson School of Medicine Pasadena, CA United States; 4 Southern California Permanente Medical Group Harbor City, CA United States; 5 The Permanente Medical Group Kaiser Permanente Northern California Oakland, CA United States; 6 Division of Research Kaiser Permanente Northern California Oakland, CA United States; 7 Genentech, a Member of the Roche Group San Francisco, CA United States

**Keywords:** natural language processing, NLP, COVID-19, symptoms, disease characterization, artificial intelligence, symptoms, application, data, cough, fever, headache, surveillance

## Abstract

**Background:**

Natural language processing (NLP) of unstructured text from electronic medical records (EMR) can improve the characterization of COVID-19 signs and symptoms, but large-scale studies demonstrating the real-world application and validation of NLP for this purpose are limited.

**Objective:**

The aim of this paper is to assess the contribution of NLP when identifying COVID-19 signs and symptoms from EMR.

**Methods:**

This study was conducted in Kaiser Permanente Southern California, a large integrated health care system using data from all patients with positive SARS-CoV-2 laboratory tests from March 2020 to May 2021. An NLP algorithm was developed to extract free text from EMR on 12 established signs and symptoms of COVID-19, including fever, cough, headache, fatigue, dyspnea, chills, sore throat, myalgia, anosmia, diarrhea, vomiting or nausea, and abdominal pain. The proportion of patients reporting each symptom and the corresponding onset dates were described before and after supplementing structured EMR data with NLP-extracted signs and symptoms. A random sample of 100 chart-reviewed and adjudicated SARS-CoV-2–positive cases were used to validate the algorithm performance.

**Results:**

A total of 359,938 patients (mean age 40.4 [SD 19.2] years; 191,630/359,938, 53% female) with confirmed SARS-CoV-2 infection were identified over the study period. The most common signs and symptoms identified through NLP-supplemented analyses were cough (220,631/359,938, 61%), fever (185,618/359,938, 52%), myalgia (153,042/359,938, 43%), and headache (144,705/359,938, 40%). The NLP algorithm identified an additional 55,568 (15%) symptomatic cases that were previously defined as asymptomatic using structured data alone. The proportion of additional cases with each selected symptom identified in NLP-supplemented analysis varied across the selected symptoms, from 29% (63,742/220,631) of all records for cough to 64% (38,884/60,865) of all records with nausea or vomiting. Of the 295,305 symptomatic patients, the median time from symptom onset to testing was 3 days using structured data alone, whereas the NLP algorithm identified signs or symptoms approximately 1 day earlier. When validated against chart-reviewed cases, the NLP algorithm successfully identified signs and symptoms with consistently high sensitivity (ranging from 87% to 100%) and specificity (94% to 100%).

**Conclusions:**

These findings demonstrate that NLP can identify and characterize a broad set of COVID-19 signs and symptoms from unstructured EMR data with enhanced detail and timeliness compared with structured data alone.

## Introduction

COVID-19, the infection caused by the novel coronavirus, SARS-CoV-2 [1], has accounted for more than 623 million cases and more than 6.5 million deaths globally as of October 2022 [2]. SARS-CoV-2 primarily affects the respiratory system but can also affect the cardiovascular, gastrointestinal, neurologic, and other systems [3-6]. The most common signs and symptoms include fever, cough, shortness of breath, fatigue, muscle aches, headaches, loss of taste or smell, sore throat, congestion, nausea or vomiting, and diarrhea [7]. However, prevalence estimates for each sign or symptom have been inconsistent, with most being derived from studies relying on self-reported surveys that are more subjective than electronic medical records (EMR) [4,8,9]. Of the studies using EMR for disease characterization, most are restricted to subgroups of patients (ie, hospitalized patients) who may have distinct symptom profiles [3,10,11]. An improved understanding of signs and symptoms of COVID-19 can inform patient care and improve population screening and disease surveillance.

Signs and symptoms can be documented in EMR by health care providers in four primary forms, broadly defined as “structured” and “unstructured,” which are as follows: (1) structured COVID-19 lab test order–related questionnaires; (2) structured diagnosis codes; (3) structured clinical notes (which may include self-reported information); and (4) unstructured free-text clinical notes. However, of the few large-scale studies using EMR, most are limited to structured data alone, particularly International Classification of Diseases (ICD) diagnoses, which have demonstrated low concordance with self-reported information due to incomplete documentation during physician visits [12]. Natural language processing (NLP) is a subfield of artificial intelligence devoted to the understanding and generation of language and can be used to supplement structured data fields with data extracted from unstructured health care provider notes across different EMR data sources [13]. In short, NLP algorithms can be designed to convert information residing in natural language into structured formats for medical research, public health surveillance, and clinical decision support [14]. During the COVID-19 pandemic, NLP has mostly been used to extract key information on COVID-19 from scientific publications [15], media articles [16], or social media platforms [17]. However, despite containing rich information on signs and symptoms of COVID-19, limited NLP-based tools have been developed for COVID-19 information extraction from unstructured EMR data. The highest-quality study thus far used an NLP-based tool termed “COVID-19 SignSym” to extract signs or symptoms from a small subset of clinical notes and performed a small validation study using data collected from 3 institutions in the United States [18]. However, the real-world application and overall usefulness of NLP for this purpose has not been assessed at scale in a large population.

Large integrated health care systems with access to complete EMR data provide a unique resource to investigate the value of NLP algorithms in the extraction of additional information from unstructured text fields. This paper describes the distribution and time of the onset of COVID-19 signs and symptoms before and after supplementing structured EMR with an NLP algorithm among more than 350,000 members of a large integrated health care system. In addition, we performed a validation substudy to assess the accuracy of the NLP algorithm in identifying COVID-19 signs and symptoms.

## Methods

### Study Setting

Kaiser Permanente Southern California (KPSC) is one of the largest integrated health care systems in the United States providing medical services to over 4.7 million members. KPSC’s comprehensive EMR data contains individual-level structured data (including diagnosis codes, procedure codes, self-assessment health forms, medications, immunization records, and laboratory results) and unstructured data (including free-text clinical notes, radiology reports, and pathology reports) covering all medical visits. Therefore, the EMR represents a standardized data collection method across all health care settings (ie, all outpatient services, hospitals, emergency department, and virtual care encounters). Care delivered to members outside of the KPSC system is also captured, as outside providers must submit detailed claims to KPSC for reimbursement. KPSC has a diverse member population that is largely representative of all residents in Southern California with health insurance [19]. As of December 2018, persons of Hispanic or Latino race or ethnicity make up the largest proportion of KPSC members (43%), followed by Non-Hispanic White (35%), Non-Hispanic Asian or Pacific Islander (12%), Non-Hispanic Black or African American (9%), and Other (1%).

### Study Population

This is a retrospective cohort study of KPSC patients of all ages with positive SARS-CoV-2 laboratory tests from March 2020 to May 2021. SARS-CoV-2 tests of all types (ie, PCR and antigen tests) across all care settings were included. Participants were included in the analysis if they had at least 6 months of continuous KPSC membership (allowing for a 45-day administrative enrollment gap between memberships) prior to the date of their first positive COVID-19 test.

### Signs or Symptoms of COVID-19

All EMR records were searched for 12 prespecified signs and symptoms within 30 days prior to and following the positive COVID-19 lab test order date. Signs and symptoms included fever, cough, headache, fatigue, dyspnea, chills, sore throat, myalgia, anosmia, diarrhea, vomiting or nausea, and abdominal pain, consistent with the Centers for Disease Control and Prevention (CDC) definitions [7,20]. If none of the above signs or symptoms were detected in the EMR, the patient was categorized as asymptomatic. Signs or symptoms were identified from the following three primary sources in the EMR: (1) ICD-10 diagnosis codes; (2) keywords or phrases in medical charts; or (3) COVID-19 lab order–related questionnaires. Keywords for signs and symptoms were predetermined in consultation with trained clinicians. The complete list of ICD-10 diagnosis codes and keywords or phrases used to identify signs and symptoms can be found in Table S1 in Multimedia Appendix 1.

### NLP Algorithm Development

**Figure 1:**
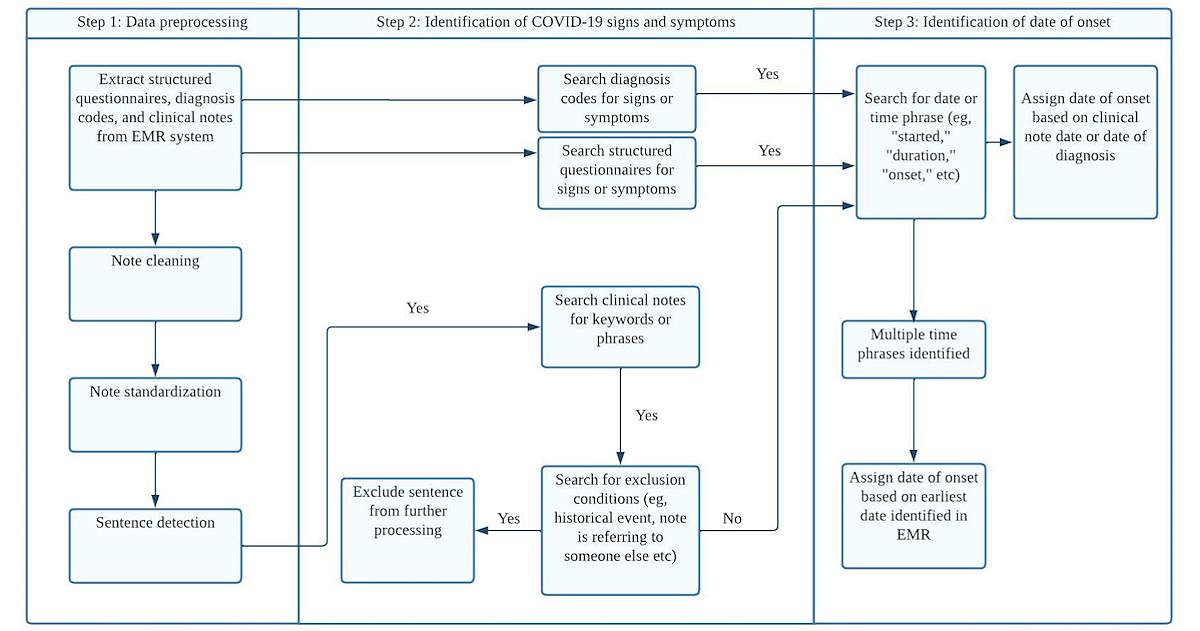
Flow diagram describing the natural language processing algorithm for detecting signs and symptoms of COVID-19. EMR: electronic medical records.

An NLP algorithm was developed to identify signs and symptoms of COVID-19 and to determine their corresponding onset dates from the EMR. The algorithm development process was implemented using a rule-based approach via Python 3.6 (Python Software Foundation). This was an iterative process in which the developed algorithm was refined to align with the reference standards derived through medical chart review and adjudication. The stages of NLP algorithm development are described below and summarized in Figure 1.

#### Step 1: Data Preprocessing

Clinical notes and structured data (diagnosis codes and symptom related questionnaires) within 30 days prior to or following the order date of the positive SARS-CoV-2 lab test were extracted from the KPSC EMR system. The extracted clinical notes were preprocessed through letter lowercase conversion, misspelled word correction, abbreviated word standardization, sentence separation, and tokenization (ie, segmenting text into linguistic units such as words and punctuation) [13].

#### Step 2: Identification of Signs and Symptoms

Patients were categorized as “Yes” for a particular symptom of interest under a set of prespecified situations (eg, if EMR notes contained a keyword or phrase related to a sign or symptom of interest, or if the patient answered “Yes” to a KPSC-administered medical questionnaire regarding COVID-19 symptoms). Keywords and phrases related to the 12 symptoms of interest were compiled by searching additional diagnosis terms and ontologies in the Unified Medical Language System [21] and were enriched by experienced clinicians and the training data set. Potential variants, abbreviations, and misspellings were also identified during algorithm development and manual chart review. For example, “shortness of breath” can be abbreviated as “sob” and “nausea/vomiting” as “n/v.” Further misspellings and abbreviations are included in Table S1 in Multimedia Appendix 1. A regular expression was constructed to search and exclude sentences that contained a combination of preselected terms (eg, when notes refer to a *lack* of signs or symptoms or a *historical* medical event or indicate that signs or symptoms were experienced by someone else). A complete list of predefined sentence exclusion scenarios as well as “Yes” criteria for all signs and symptoms are provided in Table S2 in Multimedia Appendix 1.

#### Step 3: Date of Symptom Onset Determination

For each instance of identified signs or symptoms, the corresponding onset date was determined as either the clinical note date or by extracting the date from clinical notes under prespecified conditions, for example, where a date was detected with the symptom or followed with a phrase of “symptom (first) started,” “Date of symptoms (onset):,” “symptom onset date:,” and “onset:” in unstructured notes. Specific examples of prespecified conditions are included in Table S2 and Table S3 in Multimedia Appendix 1. If signs or symptoms were identified from multiple clinical notes or structured data elements, the earliest date of symptom on record was assigned as the date of onset.

### NLP Algorithm Validation

A sample of 100 randomly selected patients was used to assess the accuracy of the NLP algorithm in identifying each of the 12 signs or symptoms from unstructured EMR data, excluding patients used for the original algorithm development. Information on the presence or absence as well as the onset date of signs or symptoms were abstracted from EMR by trained chart abstractors using an abstraction manual. Patients for whom the sign or symptom complaint or onset date could not be clearly determined by the abstractors were further reviewed and adjudicated by a collaborating research physician. For this validation substudy, the manual chart review plus adjudicated results were deemed as the reference standard. The proportions of true positive, false positive, true negative, and false negative patients were used to estimate the sensitivity, specificity, positive predictive value (PPV), negative predictive value, and overall *F* score for each preselected sign or symptom of interest [22].

Sensitivity was defined as the proportion of patients correctly classified by the computerized NLP algorithm as experiencing the symptom of interest among patients identified with the sign or symptom by manual chart review. Specificity was the proportion of patients correctly classified as not experiencing the sign or symptom among individuals identified as not experiencing the sign or symptom according to chart review. PPV was the proportion of patients correctly classified as experiencing the sign or symptom of interest among those who were classified as experiencing the sign or symptom based on the NLP algorithm. Negative predictive value was the proportion of patients correctly classified as not experiencing the sign or symptom of interest among patients classified as not experiencing the sign or symptom based on the NLP algorithm. The *F* score for each comparison was calculated as (2 × PPV × sensitivity) / (PPV + sensitivity).

### Statistical Analysis

We described patient characteristics and COVID-19 symptoms by mean, SD, median, and quartiles for continuous variables, and by frequency and percentage for categorical variables. Proportions of each symptom reported using structured EMR data were compared against proportions of each symptom identified through NLP-supplemented methods. Signs and symptoms were grouped into the following four categories according to the affected body system: respiratory (cough, sore throat, and dyspnea), systemic (fever, fatigue, chills, and myalgia), gastrointestinal (diarrhea, nausea or vomiting, and abdominal pain), and neurologic (headache and anosmia). We assessed the association between characteristics of interest and inconsistencies between traditional EMR analysis using structured data and NLP supplemented analysis. All analyses were performed using Python version 3.6 and SAS statistical software version 9.4 (SAS Institute).

### Ethical Considerations

The study was reviewed by the CDC and was conducted consistent with applicable federal law and CDC policy—45 C.F.R. part 46.102(l)(2), 21 C.F.R. part 56; 42 U.S.C. Sect. 241(d); 5 U.S.C. Sect. 552a; 44 U.S.C. Sect. 3501 et seq. The study protocol was reviewed and approved by the KPSC Institutional Review Board (#12395) with a waiver of requirement for informed consent. Only authorized persons were provided access to individual-level patient data.

## Results

### Study Population

The study cohort included 359,938 patients with a positive SARS-CoV-2 laboratory test during March 2020-May 2021. Most patients were Hispanic (219,751/359,938, 61.0%), the mean age was 40.1 (SD 19.2) years, and approximately half (191,630/359,938, 53.2%) were female participants (Table 1). The most common comorbidities were hyperlipidemia (49,743/359,938, 13.8%), hypertension (48,637/359,938, 13.5%), and diabetes (41,591/359,938, 11.6%). The majority (252,869/359,938, 70.3%) of patients lived in census tracts with a median household income of less than US $80,000. Overall, 11.5% (41,307/359,938) of patients were enrolled in Medicaid.

**Table 1:** Baseline characteristics of the study population.

Characteristics		Values (N=359,938)
**Sex, n (%)**		
	Female	191,630 (53.2)
	Male	168,308 (46.8)
**Race or ethnicity, n (%)**		
	Non-Hispanic White	72,705 (20.2)
	Hispanic	219,751 (61.1)
	Non-Hispanic Black	21,541 (6.0)
	Non-Hispanic Asian	21,723 (6.0)
	Non-Hispanic Pacific Islander	2362 (0.7)
	Non-Hispanic Native American or Alaskan	639 (0.2)
	Other or unknown	21,217 (5.9)
**Age (years) at time of SARS-CoV-2 test, n (%)**		
	0-17	44,915 (12.5)
	18-64	274,932 (76.4)
	>65	40,091 (11.1)
Age (years), mean (SD)		40.4 (19.2)
Age (years), median (IQR)		40.0 (26.0, 55.0)
**BMI, kg/m^2^, n (%)**		
	<18.5	20,778 (5.8)
	18.5-24.9	72,642 (20.2)
	25.0-29.9	102,078 (28.4)
	30.0-34.9	79,394 (22.1)
	35.0-39.9	40,617 (11.3)
	40.0-44.9	17,746 (4.9)
	≥45.0	11,828 (3.3)
	Missing	14,855 (4.1)
**Tobacco use status, n (%)**		
	Current	9701 (2.7)
	Former	50,013(13.9)
	Never	226,518 (62.9)
	Unknown	73,706 (20.5)
**Comorbidities, n (%)**		
	Hyperlipidemia	49,743 (13.8)
	Hypertension	48,637 (13.5)
	Diabetes	41,591 (11.6)
	Chronic pulmonary disease	21,254 (5.9)
	Renal disease	10,298 (2.9)
	Cancer	5401 (1.5)
	Stroke	2937 (0.8)
**Median annual household income** ^a^ **(US $), n (%)**		
	<40,000	41,352 (11.5)
	40,000-79,999	211,517 (58.8)
	≥80,000	106,886 (29.7)
	Missing	183 (0.1)
**Insurance, n (%)**		
	Medicaid	41,307 (11.5)
	Medicare	36,013 (10.0)
**Calendar period of SARS-CoV-2 test, n (%)**		
	March-May 2020	9138 (2.5)
	June-August 2020	51,406 (14.3)
	September-November 2020	54,936 (15.3)
	December 2020-February 2021	233,707 (64.9)
	March-May 2021	10,751 (3.0)

^a^Measured at the census tract level.

### COVID-19 Signs and Symptoms

**Figure 2:**
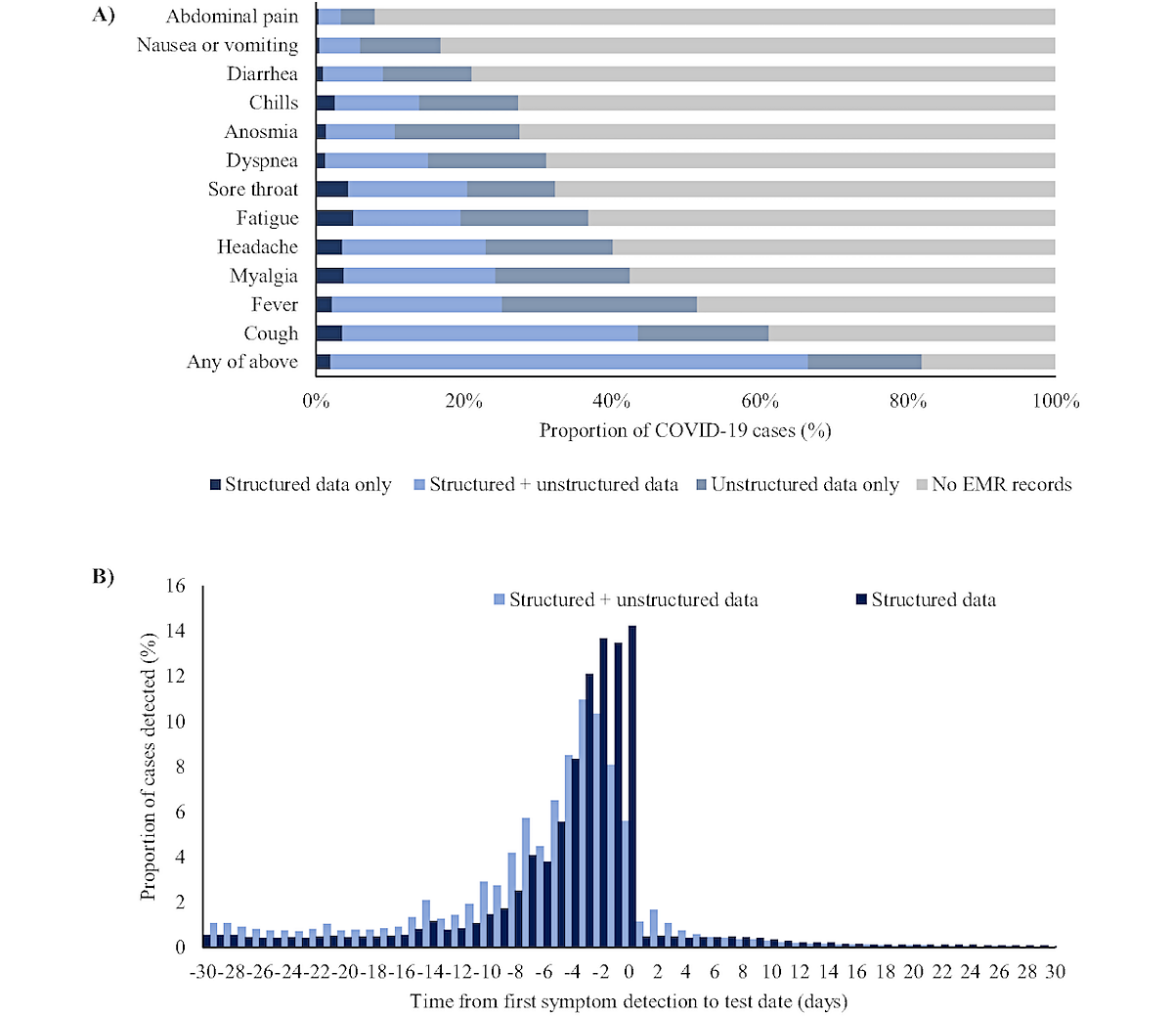
A comparison between structured and unstructured data. (A) Proportion of patients with SARS-CoV-2 with identified selected symptoms reported through structured and unstructured electronic medical records (EMR) data, by sign or symptom. (B) Days between testing and reported symptom onset before and after supplementing structured data with unstructured data (this includes IDC-10 codes, COVID-19 test-related questionnaires, and symptoms collected via keywords or phrases). ICD: International Classification of Diseases.

Supplementing structured EMR data with unstructured EMR data identified 55,568 additional symptomatic infections that were previously defined as asymptomatic based on structured data alone, representing 15.4% (55,568/359,938) of all infections. This proportion of additional identified symptomatic infections did not vary substantially by sex, age group, or race and ethnicity (Table S4 in Multimedia Appendix 1). However, there was an apparent decrease in the relative proportion of symptomatic infections identified with unstructured data during June-August 2020, whereby a higher proportion of all symptomatic cases (47,630/51,406, 92.7%) were identified via structured data compared to other time periods (60% [6456/10,751] to 80% [7336/9138]). In NLP-supplemented analyses, the symptoms ranged in frequency of reporting, from 8.0% (28,713/359,938) for abdominal pain to 61.3% (220,631/359,938) for cough. After cough, the most common symptoms identified in EMRs using NLP-supplemented analyses were fever (185,618/359,938, 51.6%), myalgia (154,042/359,938, 42.5%), headache (144,705/359,938, 40.2%), and fatigue (132,834/359,938, 36.9%; Figure 2A). NLP-supplemented analyses identified persons reporting each symptom that otherwise would not have been identified using structured data alone. For example, the proportion of SARS-CoV-2–positive persons reporting nausea and vomiting more than doubled, from 6.1% (21,981/359,938) in analysis restricted to structured data to 16.9% (60,865/359,938) in analyses supplementing this with NLP-derived fields from unstructured data.

NLP-supplemented analyses consistently identified additional signs and symptoms across all body systems relative to structured data alone, increasing the proportion of all SARS-CoV-2–positive patients identified with respiratory symptoms from 52.6% (189,146/359,938) to 69.4% (249,987/359,938), systemic symptoms from 44.4% (159,934/359,938) to 68.9% (247,988/359,938), neurological symptoms from 29.5% (106,243/359,938) to 52.1% (187,649/359,938), and gastrointestinal symptoms from 14.8% (53,193/359,938) to 31.4% (113,006/359,938; Table 2).

Among all 359,938 patients with positive SARS-CoV-2 results, 64,633 (18%) were not identified as symptomatic at any point over the study period based on the 12 preselected symptoms used in NLP-supplemented analyses (Table 2). Among all patients identified as reporting at least one symptom, the majority (252,466/295,305, 85.5%) were tested for SARS-CoV-2 following symptom onset, and 16,491 (4.6%) were tested on the same day as symptoms were reported (Table 2). Of the remaining 26,348 persons who reported symptoms after the SARS-CoV-2 test date, most (17,956/26,348, 68.1%) reported symptoms within the first 1-7 days following the SARS-CoV-2 test. Compared with structured data alone, NLP-supplemented analyses approximately doubled the proportion of identified symptomatic cases in the 6 to 30 days prior to SARS-CoV-2 sample collection (Figure 2B). The median time between the onset of first symptom and obtaining a test for SARS-CoV-2 was 3 days (IQR 1-6) for analysis restricted to traditional structured EMR data, and 4 days (IQR 2-9) for analysis supplemented with NLP algorithms.

NLP-supplemented analyses also increased the number of signs or symptoms identified per individual, often across multiple body systems. The proportion of patients reporting greater than 4 symptoms more than doubled in NLP-supplemented analysis compared to structured data alone, from 25.1% (90,202/359,938) to 53.1% (190,961/359,938) of all cases (Table 2). Similarly, the proportion of patients reporting symptoms related to 3 or more body systems increased from 22.6% (81,229/359,938) to 49.3% (177,440/359,938) after applying the NLP algorithm.

**Table 2:** COVID-19 characterization within 30 days prior to and after SARS-CoV-2 test date among all patients with confirmed SARS-CoV-2 infection (N=359,938), by data type.

Characteristics		Structured data	Structured and unstructured data
**Days between testing and symptom onset^a^, n (%)**			
	Testing 15-30 days after symptom onset	19,376 (5.4)	42,696 (11.9)
	10-14 days after onset	12,751 (3.5)	28,317 (7.9)
	7-9 days after symptom onset	19,896 (5.5)	37,325 (10.4)
	4-6 days after symptom onset	42,368 (11.8)	57,569 (16.0)
	1-3 days after symptom onset	94,157 (26.2)	86,559 (24.1)
	Tested on same day as symptom onset	34,146 (9.5)	16,491 (4.6)
	1-7 days before symptom onset	7949 (2.2)	17,956 (5.0)
	8-14 days before symptom onset	5053 (1.4)	5147 (1.4)
	15-30 days before symptom onset	4041 (1.1)	3245 (0.9)
	No symptoms reported	120,201 (33.4)	64,633 (18.0)
Days between testing and symptom onset^a^, mean (SD)		–3.96 (7.46)	–6.31 (8.49)
Days between testing and symptom onset^a^, median (IQR)		–3.00 (–6.00, –1.00)	–4.00 (–9.00, –2.00)
**Number of symptoms reported^a^, n (%)**			
	None	120,201 (33.4)	64,633 (18.0)
	1-3	149,535 (41.5)	104,344 (30.0)
	4-6	72,929 (20.3)	111,132 (30.9)
	7-9	16,164 (4.5)	65,037 (18.1)
	10-12	1109 (0.3)	14,792 (4.1)
**Body system Involved^a,b^, n (%)**			
	Respiratory	189,146 (52.6)	249,987 (69.4)
	Gastrointestinal	53,193 (14.8)	113,006 (31.4)
	Systemic	159,934 (44.4)	247,988 (68.9)
	Neurologic	106,243 (29.5)	187,649 (52.1)
**Number of body systems involved^a^, n (%)**			
	No symptoms reported	120,201 (33.4)	64,633 (18.0)
	1	70,399 (19.6)	41,452 (11.5)
	2	88,109 (24.5)	76,413 (21.2)
	3	63,017 (17.5)	105,408 (29.3)
	4	18,212 (5.1)	72,032 (20.0)

^a^Within 30 days prior to and after SARS-CoV-2 test date.

^b^Reported the percentage among the study cohort for each body system.

### NLP Algorithm Validation

Compared to signs or symptoms identified using structured data only, NLP-supplemented analyses consistently returned a high proportion of true positive cases across the signs and symptoms studied, with PPV values of >95% for all symptoms except abdominal pain (75%). Sensitivity ranged from 87% for nausea or vomiting to 100% for cough, fever, anosmia, and abdominal pain (Table 3). Specificity ranged from 94.1% for chills to 100% (7 symptoms). *F* scores ranged from 0.86 to 1.00, with the majority being over 0.90. Regarding validation of onset time, 87% of onset dates identified by NLP were within +/- 3 days of those found by chart review; 70% were the same date (Table S5 in Multimedia Appendix 1).

**Table 3:** Performance measurements of natural language processing (NLP) algorithm to identify COVID-19 signs or symptoms, as compared with chart-confirmed validation data.

Sign or symptom	Chart review, (n/N)	TP^a^ by NLP	TN^b^ by NLP	FN^c^ by NLP	FP^d^ by NLP	Sensitivity^e^ (%)	Specificity^f^ (%)	PPV^g^ (%)	NPV^h^ (%)	*F* score^i^
Cough	76/100	76	23	0	1	100.0	100.0	98.7	95.8	1.00
Fever	73/100	73	23	0	4	100.0	100.0	94.8	85.2	0.97
Body ache	67/100	64	33	3	0	95.5	100.0	100.0	91.7	0.98
Headache	54/100	50	46	4	0	92.6	100.0	100.0	92.0	0.96
Fatigue	48/100	44	50	4	2	91.7	96.2	95.7	92.6	0.94
Dyspnea	40/100	38	60	2	0	95.0	100.0	100.0	96.8	0.97
Sore throat	49/100	46	51	3	0	93.9	100.0	100.0	94.4	0.97
Anosmia	35/100	35	65	0	0	100.0	100.0	100.0	100.0	1.00
Chills	36/100	32	64	4	0	88.9	94.1	100.0	100.0	0.94
Diarrhea	29/100	28	70	1	1	96.6	98.6	96.6	98.6	0.97
Nausea or vomiting	23/100	20	76	3	1	87.0	98.7	95.2	96.2	0.91
Abdominal pain	9/100	9	88	0	3	100.0	96.7	75.0	100.0	0.86

^a^TP: true positive.

^b^TN: true negative.

^c^FN: false negative.

^d^FP: false positive.

^e^The proportion of symptoms correctly classified by the computerized algorithm (TP) among all cases (TP+FN) ascertained by chart review.

^f^The proportion of cases correctly classified as absence of symptoms by the computerized algorithm (TN) among all individuals without symptom (TN+FP) according to chart review.

^g^PPV: positive predictive value—the proportion of symptom cases correctly classified (TP) among all those classified by the computerized algorithm (TP+FP).

^h^NPV: negative predictive value—the proportion of cases correctly classified as nonsymptom (TN) among all nonsymptom cases classified by the computerized algorithm (TN+FN).

^i^The overall accuracy of NLP algorithm in identifying each sign or symptom calculated as (2×PPV×sensitivity)/(PPV+sensitivity).

## Discussion

### Overview

Among more than 350,000 patients, this paper demonstrates that NLP algorithms can be used to extract unstructured data from EMR on COVID-19 signs and symptoms with enhanced detail and timeliness compared with structured data alone. To the authors’ knowledge, this analysis represents the largest population study to date using NLP-based methods for identification and characterization of COVID-19 signs and symptoms.

### Principal Findings

Overall, we observed that up to 60% of information on signs and symptoms may only be documented in the clinical narrative; however, this proportion varied widely between the conditions studied. Hence, previous real-world population studies that were limited to classical epidemiological methods (ie, using structured EMR data alone) may have underestimated the complexity and diversity of COVID-19 symptoms. This finding has important implications for patient care by improving our understanding of the whole spectrum and pathophysiology of COVID-19. This appeared particularly relevant for respiratory and gastrointestinal symptoms, whereby our data indicate that a significant proportion of symptomatic patients (24% and 53%, respectively) are overlooked when data are limited to structured components alone.

### Comparison With Prior Work

Prior studies have noted similar improvements in COVID-19 case detection when clinical notes, ICD-10 diagnosis codes, and temperature fields have been used together, particularly for gastrointestinal conditions, rash or fever, and influenza-like illness syndromes, reporting almost double the sensitivity of detection [23,24]. The highest-quality evidence describing COVID-19 signs and symptoms to date has been derived from large meta-analyses that combine data from different study populations. In a large-scale meta-analysis including EMR data from over 4.5 million patients diagnosed with COVID-19 across 23 real-world health care databases [25], of the 6 signs or symptoms studied, cough, fever, and dyspnea were the most commonly identified. In general, this pattern was similar to the results presented in this paper; however, the proportions reported per symptom were significantly lower than those identified in this study with NLP-supplemented analyses. For example, whereas 32% was the highest proportion of patients identified with a cough in the large meta-analysis, this study identified a total of 61% with cough in NLP-supplemented analyses.

Compared to a systematic review including EMR and self-reported symptom data pooled from 24,410 cases across 148 studies in 9 countries [10], we identified similar estimates for some signs and symptoms in this paper using NLP-supplemented analyses, such as cough (61% in this study vs 57%, respectively), fatigue (37% vs 31%), and anosmia (28% vs 25%). However, we observed a higher proportion of cases reporting most other prespecified symptoms, including dyspnea (31% vs 23%), sore throat (32% vs 12%), diarrhea (21% vs 10%), nausea or vomiting (17% vs 10%), abdominal pain (8% vs 4%), and headache (40% vs 13%). Importantly, gastrointestinal symptoms are increasingly being recognized as part of the COVID-19 spectrum, yet prior meta-analyses underestimate their prevalence compared with our work. One meta-analysis of 47 studies estimated diarrhea and nausea or vomiting in 7.7% and 7.8% patients with COVID 19 infection, respectively [26], and another analysis of 78 studies estimated a weighted pooled prevalence of 12.4% (95% CI, 8.2% to 17.1%) for diarrhea, 9.0% (95% CI, 5.5% to 12.9%) for nausea or vomiting, and 6.2% (95% CI, 2.6% to 10.3%) for abdominal pain [27]. In our study, approximately 21% (75,911/359,938) of patients with confirmed SARS-CoV-2 infection reported diarrhea, 17% (60,865/359,938) reported nausea or vomiting, and 8% (28,713/359,938) reported abdominal pain, all of which are higher estimates than have been reported in previous studies. Gastrointestinal involvement has been associated with delays in diagnosis compared with patients without digestive symptoms and hence may have been overlooked previously [28,29].

The observed discrepancies between this paper and prior evidence may be the direct result of the contribution of NLP algorithms when identifying COVID-19 signs and symptoms from EMR in this study, whereas prior studies have relied on structured components of EMR alone, such as ICD-10 diagnosis codes [25]. Among survey-based studies, results may be systematically biased due to responder bias or recall bias [30,31]. Importantly, study populations contributing to large meta-analyses and systematic reviews are heterogeneous with respect to their study populations and methodologies, with some restricted to symptomatic hospitalized patients [26,27,32]. Indeed, prior EMR- and survey-based studies restricted to hospitalized cases report higher frequencies of symptom complaints compared to this study [33,34]. This paper includes structured and unstructured EMR data from all care settings among a single diverse patient population of all ages, substantially expanding the scope compared with prior work.

Together, the findings presented here demonstrate the complexity of COVID-19, which often manifests as multiple diverse signs or symptoms across different body systems. With most prior large-scale real-world studies lacking unstructured EMR data, this observation may have been overlooked previously. As well as informing clinicians to guide patient care, understanding the complete array of signs or symptoms associated with COVID-19 could enhance population-level screening efforts. In addition, we found that NLP-supplemented analyses identified an earlier date of onset of potential COVID-19 signs and symptoms compared to traditional structured EMR data. Importantly, most of the transmission occurs within the first 5 days after symptom onset [35]. Therefore, by possibly facilitating identification of an earlier date of onset relative to test positivity at the population level, NLP methods could enhance public health surveillance systems, potentially informing preventive strategies to reduce community transmission.

### Limitations

This study has at least 5 limitations, some of which are ubiquitous and unavoidable in observational research. First, while we capture symptoms occurring within 30 days of a COVID-19–positive test, it is possible that the reported symptoms detected in the EMR were due to other causes. However, chart review verified that the identified symptoms occurring within 20 days of testing were attributable to COVID-19 in the overwhelming majority of cases. Nevertheless, a comprehensive assessment of the overall usefulness of NLP would have involved a comparison with symptom reports in a SARS-CoV-2–negative population. Second, SARS-CoV-2 diagnostic tests were restricted to certain populations at differing points over the study period corresponding to periods of limited availability. As such, our estimates largely represented patients with symptomatic COVID-19 who sought medical care, and therefore it is likely that asymptomatic individuals were underrepresented in our analysis. Third, we defined symptomatic COVID-19 according to 12 conditions established as signs or symptoms of COVID-19 in the scientific literature; hence, it is possible that symptomatic cases reporting conditions outside of this established list are not counted as symptomatic. Fourth, the validation data set used in this paper included a relatively small sample size, which may have led to spurious findings. However, despite the small sample, the NLP algorithm performed well when identifying COVID-19 symptoms, producing similar sensitivity, *F* statistics, and PPV values to previously developed algorithms for symptom identification and COVID-19 characterization [18,36,37]. Lastly, this study was limited to insured individuals residing in Southern California from March 2020 to May 2021. Therefore, the findings may not be representative of or generalizable to other populations or to infections attributable to SAR-CoV-2 variants such as Delta or Omicron. However, the findings reported in this paper remain internally valid over the study period in demonstrating the overwhelming advantage of applying NLP to EMR for enhanced disease characterization across multiple clinical conditions.

### Conclusions

This paper demonstrates that NLP can identify and characterize a broad set of COVID-19 signs and symptoms from medical records, with enhanced detail and timeliness, compared with prior EMR-based studies. These findings provide clear evidence that structured EMR data alone are incomplete for symptom capture, and NLP can enhance our understanding of the whole spectrum of disease pathophysiology. Further, as a scalable and timely method for disease characterization, NLP could strengthen COVID-19 surveillance beyond conventional surveillance systems.

## Appendix

Multimedia Appendix 1 Supporting information.

## Abbreviations

CDC: Centers for Disease Control and Prevention
EMR: electronic medical records
ICD: International Classification of Diseases
KPSC: Kaiser Permanente Southern California
NLP: natural language processing
PPV: positive predictive value

## Data Availability

COVID-19 cases were obtained from Kaiser Permanente Southern California (KPSC)'s electronic medical records with approval from KPSC Institutional Review Board. The data sets analyzed during this study are not publicly available due to their confidential nature.
