# Supplementary material for: Natural Language Processing for Improved Characterization of COVID-19 Symptoms: Observational Study of 350,000 Patients in a Large Integrated Health Care System
Source: JMIR Public Health Surveill. 2022 Dec 30;8(12):e41529. doi: 10.2196/41529 (PMC9822566; doi:10.2196/41529)
Supplement: Multimedia Appendix 1 [file publichealth_v8i12e41529_app1.pdf]

TABLE S1. KEYWORDS AND DIAGNOSIS CODES USED TO IDENTIFY COMMON COVID-19 SYMPTOMS

Symptom	Keyword or Phrases*	ICD10
Fever	Fever, feverish, temperature/temp/tmax over 99°F, f/c	R50.9
Cough	Cough, coughed, coughing, tussis, tussive	R05
Chills	Chills, f/c	R50.9, R68.83
Dyspnea	Dyspnea, shortness breath, shortness of breath, short of breath, sob, labored breathing, difficulty breathing, breathing difficulty, trouble breathing, tachypnea, hyperventilation, gasping, hyperpnea, breathlessness, respiratory distress	R06.xx, J80
Sore throat	Sore throat, pharyngitis	J02.0, J02.8, J02.9
Anosmia	Anosmia, ageusia, olfactory anesthesia, taste disorder, smell disorder, smell and taste problem, trouble smelling, trouble tasking, no smell, no taste, no smell or taste, no taste or smell, no smell and taste, no taste and smell, phrase combination from the three words list "loss, lost, altered, decreased, affected, diminished, reduced, disturbed, change, abnormal, disorder, lack", "of, sense of, the sense of" and "smell, taste".	R43.0, R43.1, R43.8, R43.9
Myalgia	Body aches, body aching, body pain, body soreness, muscle aches, muscle aching, muscle pain, muscle soreness, arm aches, arm aching, arm pain, arm soreness, leg aches, leg aching, leg pain, leg soreness, myalgias, myodynia	R52, M79.10, M79.18, M79.6x
Abdominal pain	Abdominal/abdomen/abd/abd. pain, abdominal/abdomen/abd/abd. colic, abdominal/abdomen/abd/abd. cramping, abdominal/abdomen/abd/abd. cramp, abdominal/abdomen/abd/abd. tenderness, stomach pain, stomach cramping, stomach cramp, intestinal pain, belly pain, gastric pain, visceral pain, epigastric pain, upper quadrant pain, luq pain, ruq pain, lower quadrant pain, llq pain, rlq pain, suprapubic pain, periumbilical pain, colicky pain, parietal pain, stomachaches, stomach aches, pain on abdominal wall movement, pain abdominal/abd/abd., acute abdomen, abdomen acute, peritoneal tenderness, percussion tenderness, rebound tenderness	R10.0, R10.1x, R10.8xx, R10.9
Diarrhea	diarrhea, loose stools, loosing stools, watery stools, loose bowel movements, loosing bowel movements, watery bowel movements, dysentery, n/d, v/d, n/v/d	R19.7
Vomiting/nausea	vomit, vomited, vomiting, throwup, throw up, throwed up, throwing up, upchuck, upchucked, upchucking, puke, puked, puking, barf, barfed, barfing, emesis, nausea, regurgitate, hematemesis, n/v, n/d, v/d	R11.xx
Fatigue	tired, tiredness, tired out, lethargic, lethargy, weary, weariness, exhausted, exhaustion, drowsiness, lassitude, listless, listlessness, feeble, sleepy, sleepiness, languor	R53.1, R53.81, R53.83
Headache	headache, migraine, hemicrania, head pain, head ache, head aching, head sore, sinus pain, phonophobia, cephalgia, temporal arteritis, sore head, aching head, ached head, sore on head, pain on head, ache on head	R51.x, G43.0xx, G43.1xx, G43.4xx, G43.5xx, G43.6xx, G43.8xx, G43.9xx

* Plural, verbs, and abbreviations were also included in searches

TABLE S2: ADDITIONAL METHODOLOGIC DETAILS ON NATURAL LANGUAGE PROCESSING ALGORITHM AT THE CLINICAL NOTE LEVEL

**Pre-specified sentence exclusion conditions**	Sentences were excluded for any of the following situations:<br>a. Sentence did not contain a symptom of interest (listed in **Appendix Table 1**).<br>b. Someone other than the patient had the symptom. For example, "my daughter has been exposed to a COVID-19 patient and had a fever one week ago".<br>c. Negated description of a symptom of interest. Examples included "patient denied sob and anosmia", "no fever and cough", etc.<br>d. Symptom description not referring to an actual clinical situation. For example, symptom appears in emergency guidelines or side effects description, etc.<br>e. Symptom associated with a historical term or a historical date. For example, patient had a history of shortness of breath and experienced body aches several times last year.
**Pre-specified Sign or Symptom = "Yes" conditions**	A symptom was classified as "Yes" for any of the following situations:<br>a. Sentence contained the KPSC questionnaire regarding COVID-19 symptoms, and the answer was "yes" for a specific symptom name. For example, "What symptoms does the patient have that are suspicious for COVID-19? Myalgias", "Do you have fever? Yes", etc.<br>b. Sentence contained a listed symptom which included a symptom of interest and marked as "Yes", "x" or "+".<br>c. Sentence contained symptom diagnosis codes in Appendix Table 1 associated with symptom terms of interest. For example, "primary encounter diagnosis fever R50.9".<br>d. Sentence contained a description of time or a specific date associated with symptom terms of interest. For example, "patient has been coughing since June 1.", "patient has fever for past 10 days", etc.<br>e. Symptom of interest appeared in the section for diagnosis, chief complaint and/or symptom. For example, "SX: abdominal pain and fever-102 temp.", "chief complaint: fever, chills, cough, sob".<br>f. Sentence contained a definite term prior to or after a study interested symptom. Examples included "positive for shortness of breath", "has fever on and off for 1 week", "had non-bloody productive cough", etc.
**Time-based conditions for determining symptom onset date**	For each instance of identified symptoms, the corresponding symptom date was determined with the following hierarchies:<br>a. If a date was detected with the symptom or followed with a phrase of "symptom (first) started", "Date of symptoms (onset):", "symptom onset date:" and "onset:", the detected date was assigned as the symptom date of corresponding symptom.<br>b. If a time phrase was detected with the symptom or followed with a phrase of "duration:", "duration in days:", "Onset/Intensity/Duration:", "symptom started", "onset:" and the time phrase contained an exact day or week, the date of corresponding symptom was assigned as the clinical note date minus the detected exact day or week.<br>c. If a time phrase was detected with the symptom or followed with a phrase of "duration:", "duration in days:", "Onset/Intensity/Duration:", "symptom started", "onset:" and the time phrase contained a week day, such as "Monday", the date of corresponding symptom was assigned as the actual date of that week day.<br>d. If a time phrase was detected with the symptom or followed with a phrase of "duration:", "duration in days:", "Onset/Intensity/Duration:", "symptom started", "onset:" and the time phrase contained a non-extract time phrase, such as "several days", the date of corresponding symptom was assigned as the clinical note date minus the assigned exact day of the detected time phrases listed in Appendix Table 3.<br>If none of above time phrases was detected with the symptom, the clinical note date was assigned as the date of corresponding symptom.

TABLE S3. TIME WINDOW PHRASES AND CORRESPONDING NUMERIC DAY/WEEK/MONTH ASSIGNMENTS

Time window phrase	Assigned day/week/month
Couple days	2 days
Few days	3 days
Several days	5 days
Couple weeks	2 weeks
Few weeks	3 weeks
Several weeks	1 month

TABLE S4. CHARACTERISTICS OF STUDY POPULATION BY SYMPTOMATIC STATUS USING UNSTRUCTURED AND STRUCTURED DATA

Characteristic	All (Column %)	No. (%) symptomatic (structured data), Row %	No. (%) symptomatic (structured and unstructured data)*, Row %
**All**	359,938	239,737	295,305
**Sex**			
Female	191,630 (53.2%)	128,685 (67.2%)	159,236 (83.1%)
Male	168,308 (46.8%)	111,052 (66.0%)	136,069 (80.9%)
**Race/ethnicity**			
Non-Hispanic White	72,705 (20.2%)	44,592 (61.3%)	56,125 (77.2%)
Hispanic	219,751 (61.1%)	151,761 (69.1%)	185,062 (84.2%)
Non-Hispanic Black	21,541 (6.0%)	14,769 (68.6%)	18,146 (84.2%)
Non-Hispanic Asian	21,723 (6.0%)	14,030 (64.6%)	17,697 (81.5%)
Non-Hispanic Pacific Islander	2,362 (0.7%)	1,574 (66.6%)	1,914 (81.0%)
Non-Hispanic Native American/Alaskan	639 (0.2%)	414 (64.8%)	508 (79.5%)
Other/unknown	21,217 (5.9%)	12,597 (59.4%)	15,853 (74.7%)
**Age at time of SARS-CoV-2 test (year)**			
0-17	44,915 (12.5%)	27,198 (60.6%)	34,473 (76.8%)
18-64	274,932 (76.4%)	181,730 (66.1%)	224,186 (81.5%)
65+	40,091 (11.1%)	30,809 (76.9%)	36,646 (91.4%)
Mean (SD)	40.4 (19.2)	42.2 (19.3)	41.68 (19.3)
Median (Q1, Q3)	40.00 (26.00, 55.00)	42 (28, 56)	42 (27,56)
**Body Mass Index (BMI)**			
< 18.5	20,778 (5.8%)	11,012 (53.0%)	14,745 (71.0%)
18.5 - 24.9	72,642 (20.2%)	45,858 (63.1%)	57,411 (79.0%)
25.0 - 29.9	102,078 (28.4%)	68,758 (67.4%)	84,517 (82.8%)
30.0 – 34.9	79,394 (22.1%)	55,540 (70.0%)	67,320 (84.8%)
35.0 - 39.9	40,617 (11.3%)	28,675 (70.6%)	34,917 (86.0%)
40.0 - 44.9	17,746 (4.9%)	12,739 (71.8%)	15,414 (86.9%)
≥ 45.0	11,828 (3.3%)	8,680 (73.4%)	10,435 (88.2%)
Missing	14,855 (4.1%)	8,475 (57.1%)	10,546 (71.0%)
**Tobacco use status**			
Current	9,701 (2.7%)	6,643 (68.8%)	8,181 (84.3%)
Former	50,013(13.9%)	36,925 (73.8%)	44,304 (88.6%)
Never	226,518 (62.9%)	154,804 (68.3%)	190,802 (84.2%)
Unknown	73,706 (20.5%)	41,365 (56.1%)	52,018 (70.6%)
**Co-morbidities***			
Myocardial infarction	2,404 (0.7%)	1,906 (79.3%)	2,265 (94.2%)
Congestive heart failure	4,717 (1.3%)	3,901 (82.7%)	4,522 (95.9%)
Peripheral vascular disease	12,159(3.4%)	9,614 (79.1%)	11,410 (93.8%)
Cerebrovascular disease	2,937 (0.8%)	2,275 (77.5%)	2,759 (93.9%)
Chronic pulmonary disease	21,254 (5.9%)	17,267 (81.2%)	19,791 (93.1%)
Renal disease	10,298 (2.9%)	8,411 (81.7%)	9,744 (94.6%)
Metastatic tumor/Malignancy	5,401 (1.5%)	4,015 (74.3%)	4,941 (91.5%)
Other immune disease	5,452 (1.5%)	4,188 (76.8%)	4,960 (91.0%)
Hyperlipidemia	49,743 (13.8%)	37,886 (76.2%)	45,221 (90.9%)
Hypertension	48,637 (13.5%)	37,443 (77.0%)	44,379 (91.3%)
Asthma	17,993 (5.0%)	14,436 (80.2%)	16,617 (92.4%)
Organ transplant	652 (0.2%)	557 (85.4%)	633 (97.1%)
Moderate/severe liver disease	480 (0.1%)	396 (82.5%)	461 (96.0%)
Rheumatic/inflammatory disease	2,867 (0.8%)	2,291 (79.9%)	2,686 (93.7%)
Diabetes			
DM with missing hemoglobin A1c	5,949 (1.7%)	4,508 (75.8%)	5,348 (89.9%)
DM with hemoglobin A1c level < 7.5%	18,670 (5.2%)	14,330 (76.6%)	17,146 (91.8%)
DM with hemoglobin A1c level ≥ 7.5%	16,972 (4.7%)	13,708 (80.8%)	15,821 (93.2%)
**Population density per square mile**			
≤Q2 population density	179,026 (49.7%)	120,234 (67.2%)	149,719 (83.6%)
Between Q2 and Q3 population density	90,231 (25.1%)	59,777 (66.3%)	73,350 (81.3%)
> Q3 population density	90,559 (25.2%)	59,644 (65.9%)	72,131 (79.7%)
Missing	122 (0.03%)	82 (67.2%)	105 (86.1%)
**Median annual household income**			
< $40,000	41,352 (11.5%)	29,869 (72.2%)	35,654 (86.2%)
$40,000-$79,999	211,517 (58.8%)	143,090 (67.7%)	175,689 (83.16%)
≥ $80,000	106,886 (29.7%)	66,652 (62.4%)	83,802 (78.4%)
Missing	183 (0.1%)	126 (68.9%)	160 (87.4%)

*Reported the percentage of each comorbidity except for diabetes.

Characteristic	All, (Column %)	Symptomatic (structured data)*, Row %	Symptomatic (Structured and unstructured data)*, Row %
**All**	359,938	239,737	295,305
**Neighborhood education**			
≥50% more than high school	158,976 (44.2%)	110,478 (69.5%)	134,428 (84.6%)
<50% more than high school	200,831 (55.8%)	129,171 (64.3%)	160,764 (80.1%)
Missing	131 (0.04%)	88 (67.2%)	113 (86.1%)
**Influenza Vaccination in Past 3 Years**			
Yes	259,365 (72.1%)	175,817 (67.8%)	216,568 (83.5%)
No	100,573 (27.9%)	63,920 (63.6%)	78,737 (78.3%)
**Insurance**			
Medicaid	41,307 (11.5%)	28,577 (69.18%)	34,748 (84.1%)
Medicare	36,013 (10.0%)	27,837 (77.30%)	33,019 (91.7%)
**Care Setting**			
Inpatient	11,436 (3.18%)	10,346 (90.47%)	11,346 (99.21%)
ED	15,388 (4.28%)	14,715 (95.63%)	15,257 (99.15%)
Outpatient	155,171 (43,11%)	135,479 (87.31%)	146,788 (94.60%)
Other*	177,943 (49.44%)	79,197 (44.51%)	121,914 (68.51%)
**Calendar period of SARS-CoV-2 test**			
March-May 2020	9,138 (2.5%)	7,336 (80.3%)	9,062 (99.2%)
June - August 2020	51,406 (14.3%)	47,630 (92.7%)	50,077 (97.4%)
September - November 2020	54,936 (15.3%)	37,093 (67.5%)	46,865 (85.3%)
December 2020 - February 2021	233,707 (64.9%)	141,222 (60.4%)	180,831 (77.4%)
March – May 2021	10,751 (3.0%)	6,456 (60.1%)	8,470 (78.8%)

***Other care settings included virtual encounters, e-visits and online laboratory orders.

TABLE S5. AGREEMENT OF ONSET TIME BETWEEN NATURAL LANGUAGE PROCESSING AND CHART REVIEW

Symptom	Total*	15-30 days prior**	8-14 days prior	4-7 days prior	1-3 days prior	Same day	1-3 days	4-7 days	8-14 days	15-30 days
**Cough**	76	1 (1.3%)	0 (0.0%)	3 (4.0%)	11 (14.5)	44 (57.9%)	10 (13.2%)	5 (6.6%)	1 (1.3%)	1(1.3%)
**Fever**	73	1 (1.4%)	0 (0.0%)	2 (2.7%)	7 (9.6%)	52 (71.2%)	5 (6.9%)	3 (4.1%)	1 (1.4%)	2 (2.7%)
**Body ache**	64	0 (0.0%)	0 (0.0%)	0 (0.0%)	3 (4.7%)	51 (79.7%)	5 (7.8%)	3 (4.7%)	2 (3.1%)	0 (0.0%)
**Headache**	50	0 (0.0%)	0 (0.0%)	2 (4.0%)	2 (4.0%)	27 (54.0%)	9 (18.0%)	4 (8.0%)	2 (4.0%)	4 (8.0%)
**Fatigue**	44	0 (0.0%)	0 (0.0%)	0 (0.0%)	2 (4.6%)	32 (72.7%)	3 (6.8%)	6 (13.6%)	0 (0.0%)	1 (2.3%)
**Dyspnea**	38	1 (2.6%)	1 (1.6%)	1 (1.6%)	1 (1.6%)	31 (81.6%)	3 (7.8%)	0 (0.0%)	0 (0.0%)	0 (0.0%)
**Sore throat**	46	0 (0.0%)	0 (0.0%)	0 (0.0%)	2 (4.4%)	30 (65.2%)	12 (26.1%)	0 (0.0%)	1 (2.2%)	1 (2.2%)
**Anosmia**	35	0 (0.0%)	0 (0.0%)	0 (0.0%)	3 (8.6%)	26 (74.3%)	1 (2.9%)	3 (8.6%)	2 (5.7%)	0 (0.0%)
**Chills**	32	0 (0.0%)	0 (0.0%)	0 (0.0%)	2 (6.3%)	22 (68.8%)	2 (6.3%)	6 (18.8%)	0 (0.0%)	0 (0.0%)
**Diarrhea**	28	0 (0.0%)	0 (0.0%)	2 (7.1%)	1 (3.6%)	21 (75.0%)	4 (14.3%)	0 (0.0%)	0 (0.0%)	0 (0.0%)
**Nausea/Vomiting**	20	0 (0.0%)	0 (0.0%)	1 (5.0%)	1 (5.0%)	18 (90.0%)	0 (0.0%)	0 (0.0%)	0 (0.0%)	0 (0.0%)
**Abdominal Pain**	9	0 (0.0%)	1 (11.1%)	0 (0.0%)	0 (0.0%)	8 (88.9%)	0 (0.0%)	0 (0.0%)	1 (11.1%)	0 (0.0%)
**Total**	515	3 (0.6%)	2 (0.4%)	11 (2.1%)	35 (6.8%)	362 (70.3%)	54 (10.5%)	30 (5.8%)	10 (1.9%)	9 (1.7%)

*Total includes symptoms that were identified by both chart review and natural language processing

**Natural language processing identified symptom onset time 15-30 days prior to the date identified by chart review; the same logic follows for other column headings
